# Associations among different functional and structural arterial wall properties and their relations to traditional cardiovascular risk factors in healthy subjects: a cross-sectional study

**DOI:** 10.1186/1471-2261-12-29

**Published:** 2012-04-25

**Authors:** Mojca Lunder, Miodrag Janic, Natasa Kejzar, Miso Sabovic

**Affiliations:** 1 Department of Vascular Disease, University of Ljubljana Medical Centre, Zaloška 7, 1000, Ljubljana, Slovenia; 2 Institute of Pharmacology and Experimental Toxicology, Faculty of Medicine, University of Ljubljana, Korytkova 2, 1000, Ljubljana, Slovenia; 3 Institute for Biostatistics and Medical Informatics, Faculty of Medicine, University of Ljubljana, Vrazov trg 2, 1000, Ljubljana, Slovenia

## Abstract

**Background:**

The arterial wall possesses several functional and structural properties that define arterial health. Once they become impaired, cardiovascular risk increases. We aimed to ascertain the pattern of correlations among different arterial wall properties and to explore their relations to traditional risk factors and cardiovascular risk stratification. To allow such an investigation a middle-aged healthy population was recruited.

**Methods:**

This cross-sectional study included 100 healthy males (aged 41.9 ± 6.4 years). Pulse wave velocity (PWV), β-stiffness and intima-media thickness (IMT) of the carotid artery, and brachial artery flow-mediated dilation (FMD) were measured by a standardized ultrasound approach.

**Results:**

No correlation between FMD and IMT was found; only relatively poor correlations between PWV (or β-stiffness) and FMD existed, as well as between PWV (or β-stiffness) and IMT. PWV and β-stiffness highly correlated. Unexpectedly, only weak associations between PWV, β-stiffness, FMD, IMT and traditional risk factors were revealed. Hence, traditional risk factors (mainly age) explained only 10-50% of variability for PWV, β-stiffness, FMD and IMT. Although the subjects had low cardiovascular risk according to their Framingham score, their arterial wall properties were already impaired, particularly FMD.

**Conclusions:**

In healthy middle-age males we found: i) absent or poor correlations among arterial stiffness, IMT and endothelial function; ii) a low impact of traditional risk factors on the studied variables, and iii) the presence of impaired arterial wall properties despite low calculated cardiovascular risk. These results provide a deepened understanding of arterial wall properties and could help to improve cardiovascular risk stratification.

## Background

Cardiovascular diseases remain the major cause of morbidity and mortality in developed countries, atherosclerosis being the leading underlying cause [1]. Arterial functional and structural impairment, i.e. endothelial dysfunction [2], increased arterial stiffness [3] and increased intima-media thickness [4], has an important predictive value for cardiovascular events. However, they are not yet included in the generally used modes for calculation of cardiovascular risk. The most relevant functional and structural arterial wall properties are endothelial function (commonly measured by brachial artery flow-mediated dilation (FMD)), arterial stiffness (commonly measured by pulse-wave velocity (PWV), and local stiffness measurement, such as carotid artery β-stiffness or carotid distensibility) and intima-media thickness (IMT) of the carotid artery [5-8].

Calculation of cardiovascular risk is, according to current recommendations, based on the traditional risk factors of age, gender, arterial pressure, cholesterol, diabetes mellitus, smoking, etc. One of the most widely used score systems is the Framingham risk score [9,10].

Importantly, recent studies have revealed the necessity to find additional variables which would enable a more accurate cardiovascular disease risk assessment. Hence, several new potential risk markers were suggested and tested. On one hand, laboratory variables, such as high sensitivity C-reactive protein (hsCRP), homocysteine, interleukins, etc. seem to be promising [3,11,12]. On the other, selected arterial wall properties could more precisely provide insight into target organ damage, revealing the preclinical and clinical state of arterial disease and the consequent cardiovascular risk [13-15]. As mentioned above, several arterial wall properties could be measured, such as IMT, arterial wall stiffness variables and brachial artery FMD. However, the relations among them have not been clearly explored. Hence, the aim of the present study was to explore i) whether the above mentioned arterial wall properties correlate among themselves and therefore whether they all represent the same general arterial wall characteristic and/or the same underlying mechanism; ii) to what extent traditional risk factors explain or describe variations in the listed arterial wall variables and iii) whether the presence of a calculated low risk according to the Framingham score excludes the possibility of the presence of impaired arterial wall properties. To be able to answer these questions, we enrolled a healthy middle-aged male population.

## Methods

### Participants

One hundred healthy middle-aged males were recruited in this cross-sectional study. Inclusion criteria were male gender, age between 30 and 50 years, while the exclusion criteria comprised of a history of arterial hypertension, hypercholesterolemia, diabetes, smoking and other clinical cardiovascular diseases or a history of any other chronic or acute diseases. Regular medication therapy was also regarded as an exclusion criterion. The National Medical Ethics Committee of Slovenia approved the study and informed consent was obtained from all participants.

### Study design

Participants enrolled in the study after an at least 8 hours fast and 12 hours of caffeine and alcohol abstinence. They were asked not to perform intensive physical activity for at least 24 hours prior to the measurements. Upon arrival, a complete medical history was taken and a full medical examination was performed. Participants were then asked to rest in a supine position for approximately 10 minutes in order to reach acclimatisation with the environment (standardized conditions, quiet atmosphere, temperature maintained at 24°C). After acclimatisation, ultrasound measurements were performed, comprising brachial artery FMD, determination of common carotid artery IMT and stiffness variables (PWV and β-stiffness). Brachial blood pressure was recorded with an automated sphygmomanometer

(Welch Allyn Speidel & Keller OSZ Digital Blood Pressure System). However, the measurement of local pressure in carotid artery by using applanation tonometry would be more appropriate according to the guidelines [16]. A single examiner performed all the ultrasound measurements, using an Aloka ProSound Alpha 10 echo machine, incorporating a high-resolution eTracking system. The inter-observer variability was eliminated, since a single examiner performed all the ultrasound measurements. Intra-observer variability of FMD was 94 ± 2 % in our laboratory. Fasting venous blood samples were also taken. The Framingham risk score was calculated for each of the participants.

### Ultrasound measurements of selected arterial wall properties

Endothelial function was measured by means of right brachial artery FMD in accordance with the guidelines [17,18]. Participants lay in a supine position, extending their right arm on a foam cushion. A pressure cuff was placed around their right forearm. Following the visualisation of the right brachial artery, the echo machine performed continuous tracking and recording of its diameter. Baseline diameter was assessed for 1 minute, and then the forearm blood pressure cuff was inflated to 50 mmHg above the systolic pressure, producing an occlusion for a period of 4 minutes, followed by rapid cuff deflation, inducing reactive hyperaemia. Brachial artery diameter was then recorded for another 3 minutes. When the resting brachial artery diameter was reached, nitroglycerine-induced, endothelium-independent brachial artery dilation was measured following sublingual application of nitroglycerine (0.8 mg). The echo machine provided the FMD and nitroglycerine-dependent values automatically.

Ultrasound measurements of IMT, PWV and β-stiffness were performed on the common carotid artery in participants lying supine. Intima-media thickness was determined as the mean of three consecutive thickness measurements. The Aloka echo machine incorporated eTracking software for automatic arterial stiffness variables determination. After noninvasively obtaining pressure waveforms using arterial diameter change, automatic calibration of the waveforms was executed, based on the values of systolic and diastolic blood pressure. Pulse wave velocity and β-stiffness were then automatically calculated, as a mean of twelve beats.

### Blood tests

Blood samples were collected for measurement of fasting blood glucose, total cholesterol, high-density lipoprotein (HDL) cholesterol, low-density lipoprotein (LDL) cholesterol and triglycerides in serum. The above-mentioned variables were determined using a VITROS 5,1FS Chemistry system (Ortho Clinical Diagnostics, Inc.).

### Framingham risk score calculation

Framingham risk score was calculated for every participant by the classical Framingham equation. The ten-year incidence of coronary heart disease was taken as an endpoint. The risk factors age, gender, systolic blood pressure, arterial pressure, smoking, diabetes mellitus, total and HDL cholesterol concentrations or body mass index (BMI) were included in the score calculation.

### Data analysis

As the functional and morphological variables, as well as traditional risk factors were not all normally distributed, Spearman’s rank correlation coefficient was computed to assess the correlations among them. We were limited by an actual sample size of 100 so that the sample size could not be based on the statistical power calculation. Nevertheless we report the sensitivity power calculation and 95 % confidence intervals to give an idea of the potential size of the correlations. For our sample size we expect to have an 80% statistical power when detecting a correlation of size 0.3 (levels were corrected for the number of hypotheses tested by the Holm-Bonferroni method). The 95% confidence intervals for Spearman’s rank correlation coefficients [19] were also corrected for multiple testing.

An exploratory multiple multivariate regression model was used to identify associations between the functional and structural variables and traditional cardiovascular risk factors. Our sample size (levels corrected for multiple testing by the Bonferroni method, statistical power 80%) was sufficient to detect an explained variance (R^2^) of size 0.18. All statistical analyses were performed using the R environment [20]. Statistical power analyses were done using G*Power [21]. A statistical confidence α level of 0.05 was considered.

## Results

### Subject characteristics

Table 1 summarises the characteristics of the subjects recruited for the study.

**Table 1:** Characteristics of the 100 male subjects enrolled in the study

**Characteristic**	**Value**
***General characteristics***	
Age (years)	41.9 ± 6.4
BMI (kg/m^2^)	26.5 ± 2.9
Waist circumference (cm)	93.4 ± 8.1
Systolic BP (mmHg)	124.4 ± 8.7
Diastolic BP (mmHg)	77.2 ± 7.7
Heart rate (b.p.m.)	66.9 ± 10.7
***Blood tests***	
Total cholesterol (mmol/l)	5.6 ± 1.0
LDL cholesterol (mmol/l)	3.7 ± 0.9
HDL cholesterol (mmol/l)	1.3 ± 0.3
Triglycerides (mmol/l)	1.6 ± 0.9
Serum glucose (mmol/l)	5.1 ± 0.6
***Risk assessment***	
Framingham risk score (%)	6.7 ± 3.9
***Ultrasound measurements***	
FMD (%)	2.8 ± 1.6
PWV (m/s)	5.7 ± 0.8
ß-stiffness (U)	6.7 ± 1.7
IMT (mm)	0.6 ± 0.07

Data are expressed as means ± SD. *BMI* body mass index, *BP* blood pressure, *b.p.m.* beats per minute, *LDL* low-density lipoprotein, *HDL* high-density lipoprotein, *FMD* brachial artery flow-mediated dilation, *PWV* pulse wave velocity, *IMT* intima-media thickness

The average baseline brachial artery diameter was 4.3 ± 0.4 mm and the average FMD value (defining endothelium-dependent dilation) was 2.8 ± 1.6 %. The post-ischemic (hyperemic) flow volume during FMD increased up to 5.9 ± 2.0 ml/s. The average value of nitroglycerin dependent dilation of brachial artery (defining endothelium-independent dilation) was 15.8 ± 4.9 %.

### Correlations among different functional and structural arterial wall properties

**Figure 1:**
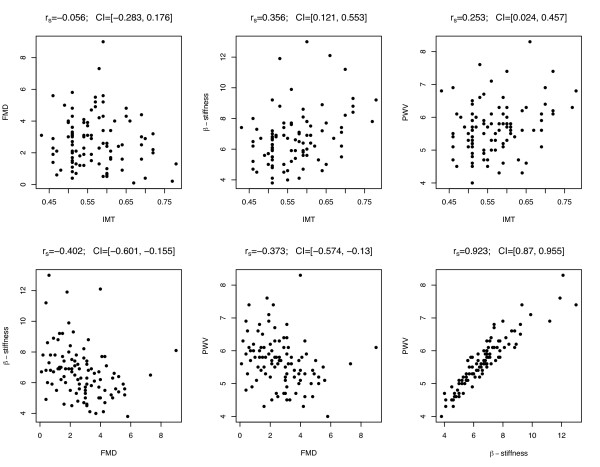
**Correlation patterns (using scatterplot representation) between functional and structural arterial wall variables (r**_**s**_**:Spearman’s rank correlation coefficient; CI: corrected 95 % confidence interval).** FMD: flow mediated dilation; IMT: intima-media thickness; PWV: pulse wave velocity

As shown in Figure 1, the strongest significant linear correlation was observed between PWV and β-stiffness. For all the correlations the 95 % confidence interval (CI) was computed (see Figure 1). A very strong significant linear correlation was observed between PWV and β-stiffness. Correlations significantly different from zero (although of moderate strength) were observed between PWV (or β-stiffness) and IMT, and between PWV (or β-stiffness) and FMD. On the other hand, no significant correlation was observed between FMD and IMT.

### Traditional risk factors and arterial wall properties

The influence of traditional risk factors on arterial wall properties was calculated using a multiple multivariate regression model. The model constructed included the majority of traditional risk factors and did not take the correlations of predictors into account (plots of residuals supported the fit of the model):

(1) $$\begin{array}{c} Arterial.wall.property=\\ =\beta_{0}+\beta_{1}\cdot Age+\beta_{2}\cdot BMI+\beta_{3}\cdot Systolic.BP+\beta_{4}\cdot Diastolic.BP\\ +\beta_{5}\cdot Heart.rate+\beta_{6}\cdot Total.cholesterol+\beta_{7}\cdot HDL.cholesterol\\ +\beta_{8}\cdot Serum.glucose+\varepsilon \end{array}$$

Due to the nature of the study the regression model was considered only as an exploratory statistical tool. Its aim was to reveal the likely associations between arterial wall properties and traditional risk factors in the population of healthy middle-aged men. A surprisingly weak link was found between traditional risk factors and the selected arterial wall properties (Table 2). We are aware of the fact that the low range of risk factors (due to sampling healthy middle-aged men) might decrease the possibility of detecting significant associations with the response variable. Therefore Table 2 shows the values of explained variance and the coefficients (with SE) of every predictor for each response variable (arterial wall property). Because of the insufficient statistical power, further explanations obtained by multivariate regressions are limited to the arterial wall properties β-stiffness, PWV and IMT.

**Table 2:** Traditional risk factors in association with arterial wall properties

	**IMT**	**FMD**	**β-stiffness**	**PWV**
Age	0.003 (0.001)*	−0.045 (0.029)	0.128 (0.025)**	0.052 (0.010)**
BMI	0.005 (0.003)	−0.022 (0.063)	0.155 (0.054)**	0.058 (0.022)*
Systolic BP	0.002 (0.001)	0.037 (0.025)	−0.012 (0.021)	−0.007 (0.009)
Diastolic BP	−0.004 (0.001)**	−0.045 (0.030)	−0.009 (0.026)	0.030 (0.011)**
HR	0 (0.001)	0.039 (0.016)*	0 (0.014)	0 (0.006)
Total Cholesterol	−0.003 (0.007)	−0.201 (0.171)	0.149 (0.147)	0.080 (0.061)
HDL Cholesterol	−0.026 (0.022)	−1.143 (0.501)*	0.808 (0.431)	0.242 (0.179)
Serum glucose	0.033 (0.012)**	−0.233 (0.275)	0.232 (0.236)	0.106 (0.098)
**Adjusted R**^**2**^	0.21	0.13	0.40	0.49

All values are multivariate linear regression coefficient values (SE). * signifies P < 0.05; ** signifies P < 0.01. Adjusted R^2^ represents the percentage of variance for each separate response variable (the last row). *BMI* body mass index, *BP* blood pressure, *HR* heart rate, *HDL* high-density lipoprotein, *FMD* brachial artery flow-mediated dilation, *PWV* pulse wave velocity, *IMT* intima-media thickness

β-stiffness was significantly positively associated with BMI and age. The model explained approximately 40 % of the variance for β-stiffness. PWV was significantly positively associated with age, diastolic blood pressure and also with BMI. The model explained approximately 49 % of variance for PWV. IMT was positively significantly associated with serum glucose levels and age, and significantly inversely associated with diastolic blood pressure. The model explained only approximately 21 % of the variance for IMT.

### Degree of impairment of arterial wall properties in respect to Framingham risk score

According to the Framingham risk score, 88 subjects included in the study had low (5.3 ± 0.3 %), while 12 subjects had moderate cardiovascular disease risk (14.1 ± 0.6 %).

FMD was reduced in 91.0 % of participants (89.0 % in the low-risk group and 100 % in the moderate-risk group) [22,23]. 8 % of participants had increased IMT of the common carotid artery (6.8 % in the low-risk group and 33.3 % in the moderate-risk group). According to the PWV and β-stiffness nomograms [24,25], 15.2 % of participants had elevated PWV (12.5 % in the low-risk group and 21 % in the moderate-risk group), while 5.9 % of participants had elevated β-stiffness (3.4 % in the low-risk group and 14 % in the moderate-risk group) adjusted for their chronological age.

## Discussion

In the present study, we found that arterial wall functional and structural properties (FMD, PWV, β-stiffness, IMT) were correlated among themselves to different degrees. Thus, there was a strong correlation between PWV and β-stiffness, but relatively weak or no correlation among the others. These results explain that widely used arterial wall variables actually correspond to entirely different arterial wall properties, those also not being inter-associated. Furthermore, we found that arterial wall properties were poorly associated with traditional risk factors, which are unable to explain a substantial part of their variation. Moreover, although most of the participants had low cardiovascular disease risk according to their Framingham score, their arterial wall properties were already impaired, the impairment being larger in a small subgroup with calculated moderate cardiovascular risk.

As mentioned, we found a high correlation between PWV and β-stiffness. This result was expected, since both variables describe the same characteristic – arterial stiffness. In the present study arterial stiffness parameters were measured locally on the carotid artery. Carotid and aortic stiffness can be used as interchangeable predictors in a low-risk population [16]. Evidently, the stiffening process, at least in its early phases, is uniformly distributed throughout the arterial tree. FMD did not correlate to IMT and relatively weakly correlated to PWV (or β-stiffness). Similarly, a relatively poor correlation existed between PWV (or β-stiffness) and IMT. It could be concluded that different processes affecting the arterial wall therefore underlie FMD, IMT and PWV (or β-stiffness). Furthermore, these different processes are seemingly neither associated nor occur in parallel, at least in the healthy population.

Investigation of the relations among different arterial wall properties was so far made in a few studies, yielding diverse results. It seems that inclusion of all parameters describing arterial stiffness, endothelial function and IMT is the only appropriate approach and that population selection importantly influences the results in the studies already performed. Obviously, the first step logically should be the complete determination of relations in a healthy population, followed in the next step by studying populations with cardiovascular diseases. Therefore, in this work we aimed to explore relations in a healthy population employing all the parameters describing arterial function and structure. This is the main difference between our study and previously performed studies. In the recent study of Koivistoinen et al young and older adults exposed to different risk factors and cardiovascular disease states were included [26]. Their results obtained on young, but not definitely healthy adults, are generally in line with our results. As in our study, a relation between PWV and carotid artery distensibility, which is basically similar to β-stiffness, was observed. The authors found that PWV did not correlate with either FMD or IMT in young adults, whereas in older adults PWV and IMT were directly and independently associated. In regard to findings obtained in young adults, we found similar results, characterized as the absence of a correlation between FMD and IMT. Interestingly, in another recent study Fitch et al reported the correlation of endothelial function (measured by digital peripheral artery tonometry) and carotid artery IMT obtained in a population of similar age [27]. These non-uniform results could be explained by the not entirely similar characteristics of the studied populations in the three studies. In distinction to the above-described studies, we included completely healthy participants. Yan *et al.* studied a large group of physically active fire-fighters. In this particular group, only the brachial artery FMD and carotid artery IMT were measured. They found, similarly to us, that these two variables did not correlate with each other [28]. It seems that disease changes the primary pattern of correlations between different variables of arterial wall properties. Overall, it seems there is enough data to conclude that the primary pattern (in young adults without cardiovascular diseases) is the lack of correlations among different arterial wall properties, whereas the presence of disease affects the properties to a degree where impaired properties start to interrelate.

The relations between traditional risk factors and arterial wall variables have been investigated in a small number of studies employing populations with small, medium or high cardiovascular disease risk, in children or in women [15,28-32]. Our results show that PWV is related to age, BMI and diastolic blood pressure, while in other studies PWV was also associated with pulse pressure, LDL concentration [15] and blood pressure [30,33]. In participants with the metabolic syndrome only elevated blood pressure was shown to be an independent factor affecting PWV [34]. The SMART study revealed associations between carotid artery IMT and its distensibility on the one hand and risk score on the other. It showed that carotid artery IMT and distensibility, which is similar to β-stiffness, were associated with overall traditional risk factors [14], while in our study β-stiffness was associated with age and BMI. Risk factors that determined IMT were age, sex, systolic blood pressure and LDL concentration [29]. The latter findings were partly similar to the results of our present study, where IMT was linked with age, diastolic blood pressure and serum glucose concentration. The association of endothelial function with triglyceride levels and associations of IMT with systolic blood pressure and smoking were reported in the study of Fitch et al [27]. In obese children, a positive correlation between BMI, systolic blood pressure and triglyceride levels and vascular variables (elastic modulus and PWV) was found [35]. In the study of Celermayer *et al.* the diminished FMD was independently associated with cigarette smoking, age and male gender in asymptomatic subjects of both sexes [31]. A significant linear correlation between FMD and LDL and HDL cholesterol concentrations was found in healthy, military men aged around 35 years [32]. The study of Yan *et al.* was also performed in a similar population of middle-aged healthy men, although their population was more physically active than ours (an average population). As in our study, traditional risk factors were only weakly associated with arterial wall variables. Only systolic blood pressure significantly inversely correlated with FMD. Age, blood pressure and LDL cholesterol concentration were independent predictors of carotid IMT. In contrast to Yan *et al.*, we also measured the additional arterial wall variables carotid artery β-stiffness and pulse wave velocity (PWV) [28]. Overall, it could be concluded that, somewhat unexpectedly, traditional risk factors explain only a small part of arterial variables in healthy young or middle-aged populations. These observations deserve further investigation.

There is a reasonable concern that calculation of the risk score solely according to traditional risk factors is not precise enough for accurate cardiovascular disease risk prediction. Therefore, in the present study we employed a population of healthy, middle-age males in order to investigate different arterial wall variables and to explore whether they might add additional value to cardiovascular risk assessment. The importance of measurement of arterial wall functional and structural properties in cardiovascular risk assessment/prediction was already examined in several clinical studies. In those studies participants of different age groups, gender, traditional risk scores and cardiovascular disease status were recruited. The authors reported that measurement of IMT and arterial distensibility improved prediction of cardiovascular disease, as the risk scores increased nearly linearly with increased IMT and decreased distensibility (measured predominantly by PWV) for patients with vascular disease, atherosclerotic risk factors and diabetes. When the predictive value of IMT and distensibility was compared, IMT appeared to better discriminate between low- and high-risk patients than distensibility [14,36]. Increased IMT was associated with increased risk of cardiovascular morbidity and mortality [37] and had the capacity to predict stroke [38]. Increased PWV is also known to be a strong predictor of cardiovascular disease [39]. Yang and colleagues found the association of increased carotid arterial stiffness only with incident ischaemic stroke but not with incident coronary heart disease [36].

Taken as a whole, the predictive value of arterial wall functional and structural properties was proven in the studies above described. However, these observations were not unanticipated since groups with moderate to high risk or evident atherosclerotic disease were studied. In these patients the risk was obviously high, demanding intensive risk factor modification and treatment. Groups who suffer atherosclerotic events in the presence of low calculated risk are definitely more challenging. Identification of patients who are at risk despite a calculated low risk is an extremely important issue, which obviously demands the inclusion of arterial wall properties in the risk calculation score. Which variables are to be included still remains an unanswered issue. Our results could be of benefit in this regard. On this point, the results of Witte *et al.* are important, since they are focused on analysing the influence of risk factors on FMD in a low-risk population [40].

There is no data available in the literature about the arterial wall condition in individuals with low risk calculated on the basis of traditional risk factors. We found that the majority of participants in our study had low cardiovascular disease risk calculated according to the Framingham score, but whose arterial wall properties were already impaired, FMD was lowered in 89.0 % of subjects of the low-risk group (setting a reference for the normal value above 6.3 % [22,23]), while PWV and β-stiffness were increased in 12.5 % and 3.4 % of subjects in the low-risk group, respectively. Even higher impairment of arterial wall properties was present in a subgroup of participants who had moderate calculated cardiovascular risk.

We believe that these results suggest that the Framingham risk score does not suffice for accurate cardiovascular disease risk prediction. Strict selection of the investigated population allows us to conclude firmly that traditional risk factors explain only a small part of the variations in arterial wall properties. It seems that in the healthy, middle-aged population, characterized by low cardiovascular risk, and consequently only slight to moderate expression of risk factors, only age contributed to vascular wall properties. Evidently, other traditional risk factors did not affect arterial wall properties significantly in this specific population group. Therefore, in this population group the addition of arterial wall properties to risk calculations such as the Framingham risk score would very likely give additional value to cardiovascular risk calculation. In our opinion, such an approach would narrow the search for subjects potentially in danger of cardiovascular events. This prediction is supported by the reported findings that survivors of myocardial infarction (independently of their actual calculated risk, which was seldom low) had diminished FMD and/or increased arterial stiffness [41,42].

This study has several limitations, as follows: a relatively small (100) number of included participants; the majority of participants included had low cardiovascular risk, and consequently normal or almost normal IMT. It is important to emphasize that we excluded participants with high risk intentionally, in order to reveal relations in the low-risk population.

Which arterial wall property adds discrimination power to risk assessment is not easy to answer. However, based on our observation that FMD, PWV and IMT primarily do not correlate, it seems more logical and meaningful to include all three variables, as opposed to only one (or two) as suggested by some authors. Furthermore, since we found a high correlation between PWV and β-stiffness, indicating that they describe the same arterial wall property, either of them could be used. Although the proposal to measure arterial wall properties in assessing risk stratification might not be globally acceptable, it would certainly improve risk stratification for participants with calculated low cardiovascular risk according to Framingham, but already possessing impaired arterial wall properties.

We believe it deserves consideration in subjects with an increased genetic burden, in subjects who have moderate risk according to traditional risk calculation and in subjects with diseases that facilitate atherosclerosis (for example rheumatoid arthritis or psoriasis).

## Conclusions

In summary, we conclude that in the population with low risk for cardiovascular diseases no or relatively weak correlations exist among different functional and structural properties of the arterial wall. We further conclude that in this population relatively poor associations exist between arterial wall variables and traditional risk factors. Taking this into account, we found that traditional risk factors in a low cardiovascular risk population explain only a small part of the variance of arterial wall properties and that the same population group could possess impaired functional and structural arterial wall properties. If in the long term these results could be translated into clinical practice, the prediction of cardiovascular risk might be improved. We suggest determination of arterial wall properties (PWV or β-stiffness, FMD and IMT) together with traditional risk factors as a potentially prospective step in future assessments, at least in selected populations.

## Competing interests

The authors declare that they have no competing interests.

## Authors’ contributions

ML participated in the study design, carried out the measurements and drafted the manuscript. MJ carried out the measurements and drafted the manuscript. NK performed the statistical analysis. MS participated in the study design, drafted the manuscript and revised it critically for important intellectual content. All authors read and approved the final manuscript.

## Funding

The present study was funded by the Slovenian Research Agency, Ljubljana Slovenia [research project L3-2293].

## Pre-publication history

The pre-publication history for this paper can be accessed here:

http://www.biomedcentral.com/1471-2261/12/29/prepub
